# An Expansion Formula with Higher-Order Derivatives for Fractional Operators of Variable Order

**DOI:** 10.1155/2013/915437

**Published:** 2013-11-10

**Authors:** Ricardo Almeida, Delfim F. M. Torres

**Affiliations:** Center for Research and Development in Mathematics and Applications (CIDMA), Department of Mathematics, University of Aveiro, 3810-193 Aveiro, Portugal

## Abstract

We obtain approximation formulas for fractional integrals and derivatives of Riemann-Liouville and Marchaud types with a variable fractional order. The approximations involve integer-order derivatives only. An estimation for the error is given. The efficiency of the approximation method is illustrated with examples. As applications, we show how the obtained results are useful to solve differential equations, and problems of the calculus of variations that depend on fractional derivatives of Marchaud type.

## 1. Introduction

Fractional calculus is a natural extension of the integer-order calculus by considering derivatives and integrals of arbitrary real or complex order *α* ∈ *𝕂*, with *𝕂* = ℝ or *𝕂* = *ℂ*. The subject was born from a famous correspondence between L'Hopital and Leibniz in 1695 and then developed by many famous mathematicians, like Euler, Laplace, Abel, Liouville, and Riemann, just to mention a few names. Recently, fractional calculus has attracted the attention of a vast number of researchers, not only in mathematics, but also in physics and in engineering, and has proven to better describe certain complex phenomena in nature [1, 2].

Since the order *α* of the integrals and derivatives may take any value, another interesting extension is to consider the order not as a constant during the process but as a variable *α*(*t*) that depends on time. This provides an extension of the classical fractional calculus and it was introduced by Samko and Ross in 1993 [3] (see also [4]). The variable order fractional calculus is nowadays recognized as a useful tool, with successful applications in mechanics, in the modeling of linear and nonlinear viscoelasticity oscillators, and in other phenomena where the order of the derivative varies with time. For more on the subject, and its applications, we mention [5–11]. For a numerical approach see, for example, [12–14]. Results on differential equations and the calculus of variations with fractional operators of variable order can be found in [15, 16] and references therein. In this paper we show how fractional derivatives and integrals of variable order can be approximated by classical integer-order operators.

The outline of the paper is the following. In Section 2 we present the necessary definitions, namely, the fractional operators of Riemann-Liouville and Marchaud of variable order. Some properties of the operators are also given. The main core of the paper is Section 3, where we prove the expansion formulas for the considered fractional operators, with the size of the expansion being the derivative of order *n* ∈ *ℕ*. In Section 4 we show the accuracy of our method with some examples and how the approximations can be applied in different situations to solve problems involving variable order fractional operators.

## 2. Fractional Calculus of Variable Order

In the following, the order of the fractional operators is given by a function *α* ∈ *C* ^1^([*a*, *b*], ]0,1[); *x*(·) is assumed to ensure convergence for each of the involved integrals. For a complete and rigorous study of fractional calculus we refer to [17].

**Definition 1**

Let *x*(·) be a function with domain [*a*, *b*]. Then, for *t* ∈ [*a*, *b*],

(i) the left Riemann-Liouville fractional integral of order *α*(·) is given by
  (1) $$\begin{array}{c} {\;}_{a}I_{t}^{\alpha (t)}x\left(t\right)=\frac{1}{\Gamma \left(\alpha \left(t\right)\right)}\int_{a}^{t}{\left(t-\tau \right)}^{\alpha (t)-1}x\left(\tau \right)d\tau , \end{array}$$
(ii) the right Riemann-Liouville fractional integral of order *α*(·) is given by
  (2) $$\begin{array}{c} {\;}_{t}I_{b}^{\alpha (t)}x\left(t\right)=\frac{1}{\Gamma \left(\alpha \left(t\right)\right)}\int_{t}^{b}{\left(\tau -t\right)}^{\alpha (t)-1}x\left(\tau \right)d\tau , \end{array}$$
(iii) the left Riemann-Liouville fractional derivative of order *α*(·) is given by
  (3) $$\begin{array}{c} {\;}_{a}D_{t}^{\alpha (t)}x\left(t\right)=\frac{1}{\Gamma \left(1-\alpha \left(t\right)\right)}\frac{d}{dt}\int_{a}^{t}{\left(t-\tau \right)}^{-\alpha (t)}x\left(\tau \right)d\tau , \end{array}$$
(iv) the right Riemann-Liouville fractional derivative of order *α*(·) is given by
  (4) $$\begin{array}{c} {\;}_{t}D_{b}^{\alpha (t)}x\left(t\right)=\frac{-1}{\Gamma \left(1-\alpha \left(t\right)\right)}\frac{d}{dt}\int_{t}^{b}{\left(\tau -t\right)}^{-\alpha (t)}x\left(\tau \right)d\tau , \end{array}$$
(v) the left Marchaud fractional derivative of order *α*(·) is given by
  (5)   a𝔻tα(t)x(t)=x(t)Γ(1−α(t))(t−a)α(t)+α(t)Γ(1−α(t))∫atx(t)−x(τ)(t−τ)1+α(t)dτ,
(vi) the right Marchaud fractional derivative of order *α*(·) is given by
  (6)   t𝔻bα(t)x(t)=x(t)Γ(1−α(t))(b−t)α(t)+α(t)Γ(1−α(t))∫tbx(t)−x(τ)(τ−t)1+α(t)dτ.

**Remark 2**

It follows from Definition 1 that

(7) $$\begin{array}{c} {\;}_{a}D_{t}^{\alpha (t)}x\left(t\right)=\frac{d}{dt}{}_{a}I_{t}^{1-\alpha (t)}x\left(t\right),\;\;\\ {\;\;}_{t}D_{b}^{\alpha (t)}x\left(t\right)=-\frac{d}{dt}{}_{t}I_{b}^{1-\alpha (t)}x\left(t\right). \end{array}$$

**Example 3 (see [3])**

Let *x* be the power function *x*(*t*) = (*t*−*a*)^*γ*^. Then, for *γ* > −1, we have

(8) aItα(t)x(t)=Γ(γ+1)Γ(γ+α(t)+1)(t−a)γ+α(t),a𝔻tα(t)x(t)=Γ(γ+1)Γ(γ−α(t)+1)(t−a)γ−α(t),  aDtα(t)x(t)=Γ(γ+1)Γ(γ−α(t)+1)(t−a)γ−α(t)−α(1)(t)Γ(γ+1)Γ(γ−α(t)+2)(t−a)γ−α(t)+1×[ln⁡⁡(t−a)−ψ(γ−α(t)+2)  +ψ(1−α(t))],

where *ψ* is the Psi function, that is, the derivative of the logarithm of the Gamma function:

(9) $$\begin{array}{c} \psi \left(t\right)=\frac{d}{dt}\ln\left(\Gamma \left(t\right)\right)=\frac{\Gamma^{\prime }\left(t\right)}{\Gamma \left(t\right)}. \end{array}$$

From Example 3 we see that _*a*_ *D*_*t*_^*α*(*t*)^ *x*(*t*) ≠ *i*_*a*_ *𝔻*_*t*_^*α*(*t*)^ *x*(*t*). Also, the symmetry on power functions is violated, when we consider _*a*_ *I*_*t*_^*α*(*t*)^ *x*(*t*) and _*a*_ *D*_*t*_^*α*(*t*)^ *x*(*t*), but holds for _*a*_ *I*_*t*_^*α*(*t*)^ *x*(*t*) and _*a*_ *𝔻*_*t*_^*α*(*t*)^ *x*(*t*). Later we explain this better, when we deduce the expansion formula for the Marchaud fractional derivative. In contrast with the constant fractional order case, the law of exponents fails for fractional integrals of variable order. However, a weak form holds (see [3]): if *β*(*t*) ≡ *β*, *t*∈]0,1[, then _*a*_ *I*_*t*_^*α*(*t*)^_*a*_ *I*_*t*_^*β*^ *x*(*t*) = _*a*_ *I*_*t*_^*α*(*t*)+*β*^ *x*(*t*).

## 3. Expansion Formulas with Higher-Order Derivatives

The main results of the paper provide approximations of the fractional derivatives of a given function *x* by sums involving only integer derivatives of *x*. The approximations use the generalization of the binomial coefficient formula to real numbers:

(10) $$\begin{array}{c} \left(\begin{array}{c} -\alpha \left(t\right)\\ k \end{array}\right){\left(-1\right)}^{k}=\left(\begin{array}{c} \alpha \left(t\right)+k-1\\ k \end{array}\right)=\frac{\Gamma \left(\alpha \left(t\right)+k\right)}{\Gamma \left(\alpha \left(t\right)\right)k!}. \end{array}$$

**Theorem 4**

Fix *n* ∈ *ℕ* and *N* ≥ *n* + 1, and let *x*(·) ∈ *C* ^*n*+1^([*a*, *b*], ℝ). Define the (left) moment of *x* of order *k* by

(11) $$\begin{array}{c} V_{k}\left(t\right)=\left(k-n\right)\int_{a}^{t}{\left(\tau -a\right)}^{k-n-1}x\left(\tau \right)d\tau . \end{array}$$

Then,

(12)   aDtα(t)x(t)=S1(t)−S2(t)+E1,N(t)+E2,N(t)

with

(13) S1(t)=(t−a)−α(t)[∑k=0nA(α(t),k)(t−a)kx(k)(t)+∑k=n+1NB(α(t),k)(t−a)n−kVk(t)],

where

(14)  A(α(t),k)1Γ(k+1−α(t)) ×[1+∑p=n+1−kNΓ(p−n+α(t))Γ(α(t)−k)(p−n+k)!],k=0,…,n, B(α(t),k)=Γ(k−n+α(t))Γ(−α(t))Γ(1+α(t))(k−n)!,S2(t)=x(t)α(1)(t)Γ(1−α(t))(t−a)1−α(t)×[ln⁡⁡(t−a)1−α(t)−1(1−α(t))2  −ln⁡⁡(t−a)∑k=0N(−α(t)k)(−1)kk+1  +∑k=0N(−α(t)k)(−1)k∑p=1N1p(k+p+1)]+α(1)(t)Γ(1−α(t))(t−a)1−α(t)×[ln⁡⁡(t−a)∑k=n+1N+n+1(  −α(t)k−n−1)  ×(−1)k−n−1k−n(t−a)n−kVk(t)  −∑k=n+1N+n+1(−α(t)k−n−1)(−1)k−n−1  ×∑p=1N1p(k+p−n)(t−a)n−k−pVk+p(t)].

The error of the approximation _*a*_ *D*_*t*_^*α*(*t*)^ *x*(*t*) ≈ *S*_1_(*t*) − *S*_2_(*t*) is given by *E*_1,*N*_(*t*) + *E*_2,*N*_(*t*), where *E*_1,*N*_(*t*) and *E*_2,*N*_(*t*) are bounded by

(15) |E1,N(t)|≤Ln+1(t)×exp⁡((n−α(t))2+n−α(t))Γ(n+1−α(t))(n−α(t))Nn−α(t)×(t−a)n+1−α(t),

(16) |E2,N(t)|≤L1(t)|α(1)(t)|(t−a)2−α(t)exp⁡(α2(t)−α(t))Γ(2−α(t))N1−α(t)×[|ln⁡(t−a)|+1N]

with

(17) $$\begin{array}{c} L_{j}\left(t\right)=\underset{\tau \in \left[a,t\right]}{\max}\left|x^{\left(j\right)}\left(\tau \right)\right|,\;j\in \left\{1,n+1\right\}. \end{array}$$

**Proof**

Starting with equality

(18) $$\begin{array}{c} {\;}_{a}D_{t}^{\alpha (t)}x\left(t\right)=\frac{1}{\Gamma \left(1-\alpha \left(t\right)\right)}\frac{d}{dt}\int_{a}^{t}{\left(t-\tau \right)}^{-\alpha (t)}x\left(\tau \right)d\tau , \end{array}$$

doing the change of variable *t* − *τ* = *u* − *a* over the integral, and then differentiating it, we get

(19)   aDtα(t)x(t)=1Γ(1−α(t))ddt∫at(u−a)−α(t)x(t−u+a)du=1Γ(1−α(t))[x(a)(t−a)α(t)+∫atddt‍[(u−a)−α(t)×x(t−u+a)]du]=1Γ(1−α(t)) ×[x(a)(t−a)α(t)+∫at[−α(1)(t)(u−a)−α(t)×ln⁡⁡(u−a)x(t−u+a)+(u−a)−α(t)×x(1)(t−u+a)]du]=S1(t)−S2(t)

with

(20) S1(t) =1Γ(1−α(t))[x(a)(t−a)α(t)+∫at(t−τ)−α(t)x(1)(τ)dτ],

(21) S2(t) =α(1)(t)Γ(1−α(t))∫at(t−τ)−α(t)ln⁡(t−τ)x(τ)dτ.

The equivalence between (20) and (13) follows from the computations of [18]. To show the equivalence between (21) and (14) we start in the same way as done in [19], to get

(22) S2(t)=α(1)(t)Γ(1−α(t))[x(t)∫at(t−u)−α(t)ln⁡(t−u)du−∫atx(1)(τ)×(∫aτ(t−u)−α(t)ln⁡(t−u)du)dτ]=α(1)(t)Γ(1−α(t))[x(t)(t−a)1−α(t)×[ln⁡(t−a)1−α(t)−1(1−α(t))2]−∫atx(1)(τ)×(∫aτ(t−a)−α(t)(1−u−at−a)−α(t)×[ln⁡⁡(t−a)+ln⁡(1−u−at−a)]du)dτ].

Now, applying Taylor's expansion over

(23) $$\begin{array}{c} {\left(1-\frac{u-a}{t-a}\right)}^{-\alpha (t)} \end{array}$$

and

(24) $$\begin{array}{c} \ln\left(1-\frac{u-a}{t-a}\right), \end{array}$$

we deduce that

(25) S2(t)=α(1)(t)Γ(1−α(t)) ×[x(t)(t−a)1−α(t)[ln⁡⁡(t−a)1−α(t)−1(1−α(t))2]−∫atx(1)(τ)(∫aτ(t−a)−α(t)ln⁡⁡(t−a)×∑k=0N(−α(t)k)(−1)k(u−a)k(t−a)kdu−∫aτ(t−a)−α(t)∑k=0N(−α(t)k)(−1)k×(u−a)k(t−a)k  ∑p=1N1p(u−a)p(t−a)pdu)dτ] +E2,N(t)=α(1)(t)Γ(1−α(t))[x(t)(t−a)1−α(t)×[ln⁡⁡(t−a)1−α(t)−1(1−α(t))2]−∫atx(1)(τ)(t−a)−α(t)ln⁡⁡(t−a)×∑k=0N(−α(t)k)(−1)k(t−a)k×(∫aτ(u−a)k  du)dτ+∫atx(1)(τ)(t−a)−α(t)×∑k=0N(−α(t)k)(−1)k(t−a)k×∑p=1N1p(t−a)p×(∫aτ(u−a)k+p  du)dτ] +E2,N(t)=α(1)(t)(t−a)−α(t)Γ(1−α(t)) ×[x(t)(t−a)[ln⁡(t−a)1−α(t)−1(1−α(t))2]−ln⁡⁡(t−a)∑k=0N(−α(t)k)(−1)k(t−a)k(k+1)×(∫atx(1)(τ)(τ−a)k+1  dτ)+∑k=0N(−α(t)k)(−1)k(t−a)k  ∑p=1N1p(t−a)p(k+p+1)×(∫atx(1)(τ)(τ−a)k+p+1dτ)]+E2,N(t).

Integrating by parts, we conclude with the two following equalities:

(26) ∫atx(1)(τ)(τ−a)k+1dτ=x(t)(t−a)k+1−Vk+n+1(t),∫atx(1)(τ)(τ−a)k+p+1dτ=x(t)(t−a)k+p+1−Vk+p+n+1(t).

The deduction of relation (14) for *S*_2_(*t*) follows now from direct calculations. Finally, we prove the upper bound formula for the error. The bound (15) for the error *E*_1,*N*_(*t*) at time *t* follows easily from [18]. With respect to sum *S*_2_, the error at *t* is bounded by

(27) |E2,N(t)|≤|α(1)(t)(t−a)−α(t)Γ(1−α(t))| ×|−ln⁡⁡(t−a)∑k=N+1∞(−α(t)  k)(−1)kk+1  ×(∫atx(1)(τ)(τ−a)k+1(t−a)k  dτ)  +∑k=N+1∞(−α(t)  k)(−1)k  ×∑p=N+1∞1p(k+p+1)  ×(∫atx(1)(τ)(τ−a)k+p+1(t−a)k+p  dτ)|.

Define the quantities

(28) $$\begin{array}{c} I_{1}\left(t\right)=\int_{N}^{\infty }\frac{1}{k^{1-\alpha (t)}\left(k+1\right)\left(k+2\right)}\;dk,\\ I_{2}\left(t\right)=\int_{N}^{\infty }\int_{N}^{\infty }\frac{1}{k^{1-\alpha (t)}p\left(k+p+1\right)\left(k+p+2\right)}\;dp\;dk. \end{array}$$

Inequality (16) follows from relation

(29) $$\begin{array}{c} \left|\left(\begin{array}{c} -\alpha \left(t\right)\\ k \end{array}\right)\right|\leq \frac{\exp\left(\alpha^{2}\left(t\right)-\alpha \left(t\right)\right)}{k^{1-\alpha (t)}} \end{array}$$

and the upper bounds

(30) $$\begin{array}{c} I_{1}\left(t\right)<\int_{N}^{\infty }\frac{1}{k^{2-\alpha (t)}}dk=\frac{1}{\left(1-\alpha \left(t\right)\right)N^{1-\alpha (t)}},\\ I_{2}\left(t\right)<\int_{N}^{\infty }\int_{N}^{\infty }\frac{1}{k^{2-\alpha (t)}p^{2}}\;dp\;dk=\frac{1}{\left(1-\alpha \left(t\right)\right)N^{2-\alpha (t)}} \end{array}$$

for *I*_1_ and *I*_2_.

Similarly as done in Theorem 4 for the left Riemann-Liouville fractional derivative, an approximation formula can be deduced for the right Riemann-Liouville fractional derivative.

**Theorem 5**

Fix *n* ∈ *ℕ* and *N* ≥ *n* + 1, and let *x*(·) ∈ *C* ^*n*+1^([*a*, *b*], ℝ). Define the (right) moment of *x* of order *k* by

(31) $$\begin{array}{c} W_{k}\left(t\right)=\left(k-n\right)\int_{t}^{b}{\left(b-\tau \right)}^{k-n-1}x\left(\tau \right)d\tau . \end{array}$$

Then,

(32) $$\begin{array}{c} {\;}_{t}D_{b}^{\alpha (t)}x\left(t\right)=S_{1}\left(t\right)+S_{2}\left(t\right)+E_{1,N}\left(t\right)+E_{2,N}\left(t\right) \end{array}$$

with

(33) S1(t)=(b−t)−α(t)[∑k=0nA(α(t),k)(b−t)kx(k)(t)+∑k=n+1NB(α(t),k)(b−t)n−kWk(t)],

where

(34) A(α(t),k)=(−1)kΓ(k+1−α(t))×[1+∑p=n+1−kNΓ(p−n+α(t))Γ(α(t)−k)(p−n+k)!],k=0,…,n,B(α(t),k)=(−1)n+1Γ(k−n+α(t))Γ(−α(t))Γ(1+α(t))(k−n)!,S2(t)=x(t)α(1)(t)Γ(1−α(t))(b−t)1−α(t) ×[ln⁡⁡(b−t)1−α(t)−1(1−α(t))2−ln⁡⁡(b−t)∑k=0N(−α(t)k)(−1)kk+1+∑k=0N(−α(t)k)(−1)k∑p=1N1p(k+p+1)]+α(1)(t)Γ(1−α(t))(b−t)1−α(t) ×[ln⁡⁡(b−t)∑k=n+1N+n+1(−α(t)k−n−1)×(−1)k−n−1k−n(b−t)n−kWk(t)−∑k=n+1N+n+1(−α(t)k−n−1)(−1)k−n−1×∑p=1N1p(k+p−n)(b−t)n−k−pWk+p(t)].

The error of the approximation _*t*_ *D*_*b*_^*α*(*t*)^ *x*(*t*) ≈ *S*_1_(*t*) + *S*_2_(*t*) is given by *E*_1,*N*_(*t*) + *E*_2,*N*_(*t*), where *E*_1,*N*_(*t*) and *E*_2,*N*_(*t*) are bounded by

(35) |E1,N(t)|≤Ln+1(t) ×exp⁡⁡((n−α(t))2+n−α(t))Γ(n+1−α(t))(n−α(t))Nn−α(t) ×(b−t)n+1−α(t),|E2,N(t)|≤L1(t)|α(1)(t)|(b−t)2−α(t)exp⁡(α2(t)−α(t))Γ(2−α(t))N1−α(t) ×[|ln⁡(b−t)|+1N]

with

(36) $$\begin{array}{c} L_{j}\left(t\right)=\underset{\tau \in \left[a,t\right]}{\max}\left|x^{\left(j\right)}\left(\tau \right)\right|,\;j\in \left\{1,n+1\right\}. \end{array}$$

Using the techniques presented in [20], similar formulas as the ones given by Theorems 4 and 5 can be proved for the left and right Riemann-Liouville fractional integrals of order *α*(·). For example, for the left fractional integral one has the following result.

**Theorem 6**

Fix *n* ∈ *ℕ* and *N* ≥ *n* + 1, and let *x*(·) ∈ *C* ^*n*+1^([*a*, *b*], ℝ). Then,

(37)   aItα(t)x(t)=(t−a)α(t)[∑k=0nA(α(t),k)(t−a)kx(k)(t)+∑k=n+1NB(α(t),k)(t−a)n−kVk(t)]+EN(t),

where

(38) A(α(t),k)=1Γ(k+1+α(t))×[1+∑p=n+1−kNΓ(p−n−α(t))Γ(−α(t)−k)(p−n+k)!],k=0,…,n,B(α(t),k)=Γ(k−n−α(t))Γ(α(t))Γ(1−α(t))(k−n)!,Vk(t)=(k−n)∫at(τ−a)k−n−1x(τ)dτ, k=n+1,…,N.

A bound for the error *E*_*N*_(*t*) is given by

(39) |EN(t)|≤Ln+1(t) ×exp⁡((n+α(t))2+n+α(t))Γ(n+1+α(t))(n+α(t))Nn+α(t) ×(t−a)n+1+α(t).

We now focus our attention on the left Marchaud fractional derivative _*a*_ *𝔻*_*t*_^*α*(*t*)^ *x*(*t*). Splitting the integral (5), we deduce that

(40) $$\begin{array}{c} {\;}_{a}{𝔻}_{t}^{\alpha (t)}x\left(t\right)=-\frac{\alpha \left(t\right)}{\Gamma \left(1-\alpha \left(t\right)\right)}\int_{a}^{t}\frac{x\left(\tau \right)}{{\left(t-\tau \right)}^{1+\alpha (t)}}d\tau . \end{array}$$

Integrating by parts,

(41)   a𝔻tα(t)x(t) =1Γ(1−α(t))[x(a)(t−a)α(t)+∫at(t−τ)−α(t)x(1)(τ)dτ],

which is a representation for the left Riemann-Liouville fractional derivative when the order is constant, that is, when *α*(*t*) ≡ *α* [17, Lemma 2.12]. For this reason, the Marchaud fractional derivative is more suitable as the inverse operation for the Riemann-Liouville fractional integral. With (41) and Theorem 4 in mind, it is not difficult to obtain the corresponding formula for _*a*_ *𝔻*_*t*_^*α*(*t*)^ *x*(*t*).

**Theorem 7**

Fix *n* ∈ *ℕ* and *N* ≥ *n* + 1, and let *x*(·) ∈ *C* ^*n*+1^([*a*, *b*], ℝ). Then,

(42) $$\begin{array}{c} {\;}_{a}{𝔻}_{t}^{\alpha (t)}x\left(t\right)=S_{1}\left(t\right)+E_{1,N}\left(t\right), \end{array}$$

where *S*_1_(*t*) and *E*_1,*N*_(*t*) are as in Theorem 4.

Similarly, having into consideration that

(43)   t𝔻bα(t)x(t) =1Γ(1−α(t))[x(b)(b−t)α(t)−∫tb(τ−t)−α(t)x(1)(τ)dτ],

the following result holds.

**Theorem 8**

Fix *n* ∈ *ℕ* and *N* ≥ *n* + 1, and let *x*(·) ∈ *C* ^*n*+1^([*a*, *b*], ℝ). Then,

(44) $$\begin{array}{c} {\;}_{t}{𝔻}_{b}^{\alpha (t)}x\left(t\right)=S_{1}\left(t\right)+E_{1,N}\left(t\right), \end{array}$$

where *S*_1_(*t*) and *E*_1,*N*_(*t*) are as in Theorem 5.

## 4. Examples

For illustrative purposes, we consider the left Riemann-Liouville fractional integral and the left Riemann-Liouville and Marchaud fractional derivatives of order *α*(*t*) = (*t* + 1)/4. Similar results as the ones presented here are easily obtained for the other fractional operators and for other functions *α*(·). All computations were done using the Computer Algebra System Maple.

### 4.1. Test Function

We test the accuracy of our approximations with an example.

**Example 1**

Let *x* be the function *x*(*t*) = *t* ^4^ with *t* ∈ [0,1]. Then, for *α*(*t*) = (*t* + 1)/4, it follows from Example 3 that

(45) $$\begin{array}{c} {\;}_{0}I_{t}^{\alpha (t)}x\left(t\right)=\frac{24}{\Gamma \left(\left(t+21\right)/4\right)\;\;}t^{(t+17)/4}, \end{array}$$

(46)   0Dtα(t)x(t)=24Γ((19−t)/4)t(15−t)/4−6Γ((23−t)/4)t(19−t)/4 ×[ln⁡(t)−ψ(23−t4)+ψ(3−t4)],

(47) $$\begin{array}{c} {\;}_{0}{𝔻}_{t}^{\alpha (t)}x\left(t\right)=\frac{24}{\Gamma \left(\left(19-t\right)/4\right)}t^{(15-t)/4}. \end{array}$$

**Figure 1:**
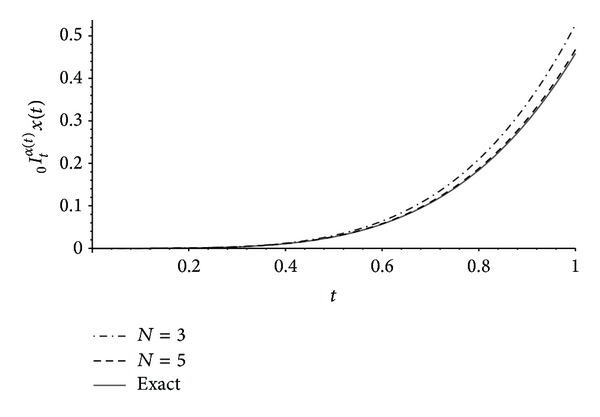
Exact (45) and numerical approximations of the left Riemann-Liouville integral _0_ *I*_*t*_^*α*(*t*)^ *x*(*t*) with *x*(*t*) = *t* ^4^ and *α*(*t*) = (*t* + 1)/4 obtained from Theorem 6 with *n* = 2 and *N* ∈ {3,5}. The error (48) is *E* ≈ 0.02169 for *N* = 3 and *E* ≈ 0.00292 for *N* = 5.

**Figure 2:**
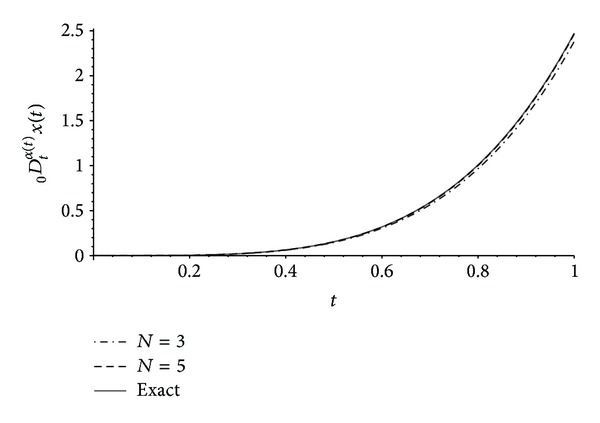
Exact (46) and numerical approximations of the left Riemann-Liouville derivative _0_ *D*_*t*_^*α*(*t*)^ *x*(*t*) with *x*(*t*) = *t* ^4^ and *α*(*t*) = (*t* + 1)/4 obtained from Theorem 4 with *n* = 2 and *N* ∈ {3,5}. The error (48) is *E* ≈ 0.03294 for *N* = 3 and *E* ≈ 0.003976 for *N* = 5.

**Figure 3:**
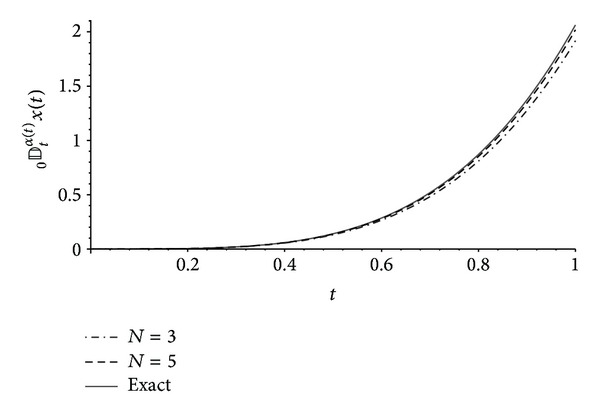
Exact (47) and numerical approximations of the left Marchaud derivative _0_ *𝔻*_*t*_^*α*(*t*)^ *x*(*t*) with *x*(*t*) = *t* ^4^ and *α*(*t*) = (*t* + 1)/4 obtained from Theorem 7 with *n* = 2 and *N* ∈ {3,5}. The error (48) is *E* ≈ 0.04919 for *N* = 3 and *E* ≈ 0.01477 for *N* = 5.

In Figures 1, 2, and 3 one can compare the exact expressions of the fractional operators of variable order (45), (46), and (47), respectively, with the approximations obtained from our results of Section 3 with *n* = 2 and *N* ∈ {3,5}. The error *E* is measured using the norm

(48) $$\begin{array}{c} E\left(f,g\right)\left(t\right)=\sqrt{\int_{0}^{1}{\left(f\left(t\right)-g\left(t\right)\right)}^{2}\;dt}. \end{array}$$

### 4.2. Fractional Differential Equations of Variable Order

Consider the following fractional differential equation of variable order:

(49) $$\begin{array}{c} {\;}_{0}{𝔻}_{t}^{\alpha (t)}x\left(t\right)+x\left(t\right)=\frac{1}{\Gamma \left(\left(7-t\right)/4\right)}t^{(3-t)/4}+t,\\ x\left(0\right)=0, \end{array}$$

with *α*(*t*) = (*t* + 1)/4. It is easy to check that $\bar{x}(t)=t$ is a solution to (49). We exemplify how Theorem 7 may be applied in order to approximate the solution of such type of problems. The main idea is to replace all the fractional operators that appear in the differential equation by a finite sum up to order *N*, involving integer derivatives only, and, by doing so, to obtain a new system of standard ordinary differential equations that is an approximation of the initial fractional variable order problem. As the size of *N* increases, the solution of the new system converges to the solution of the initial fractional system. The procedure for (49) is the following. First, we replace _0_ *𝔻*_*t*_^*α*(*t*)^ *x*(*t*) by

(50)   0𝔻tα(t)x(t)≈A(α(t),N)t−α(t)x(t)+B(α(t),N)t1−α(t)x(1)(t)+∑k=2NC(α(t),k)t1−k−α(t)Vk(t),

where

(51) A(α(t),N)=1Γ(1−α(t))[1+∑p=2NΓ(p−1+α(t))Γ(α(t))(p−1)!],B(α(t),N)=1Γ(2−α(t))[1+∑p=1NΓ(p−1+α(t))Γ(α(t)−1)p!],C(α(t),k)=Γ(k−1+α(t))Γ(−α(t))Γ(1+α(t))(k−1)!,

and *V*_*k*_(*t*) is the solution of the system

(52) $$\begin{array}{c} V_{k}^{\left(1\right)}\left(t\right)=\left(k-1\right)t^{k-2}x\left(t\right),\\ V_{k}\left(0\right)=0,\;k=2,3,\ldots ,N. \end{array}$$

Thus, we get the approximated system of ordinary differential equations

(53) [A(α(t),N)t−α(t)+1]x(t)+B(α(t),N)t1−α(t)x(1)(t)  +∑k=2NC(α(t),k)t1−k−α(t)Vk(t) =1Γ((7−t)/4)t(3−t)/4+t,Vk(1)(t)=(k−1)tk−2x(t), k=2,3,…,N,x(0)=0,Vk(0)=0, k=2,3,…,N.

**Figure 4:**
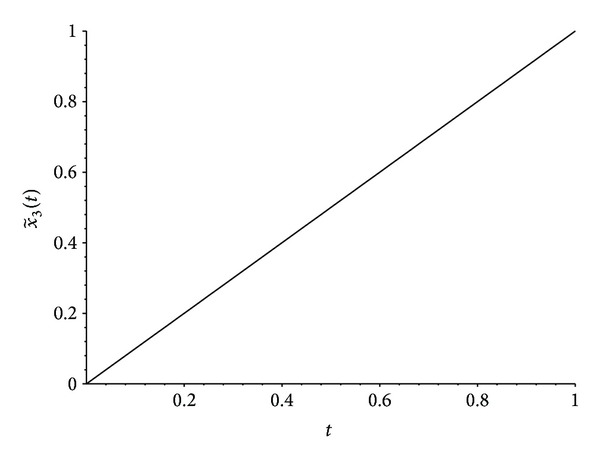
Approximation ${\tilde{x}}_{3}(t)$ to the exact solution $\bar{x}(t)=t$ of the fractional differential equation (49), obtained from the application of Theorem 7, that is, obtained by solving (53) with *N* = 3.

Now we apply any standard technique to solve the system of ordinary differential equations (53). We used the command dsolve of Maple. In Figure 4 we find the graph of the approximation ${\tilde{x}}_{3}(t)$ to the solution of problem (49), obtained by solving (53) with *N* = 3.  Table 1 gives some numerical values of such approximation, illustrating numerically the fact that the approximation ${\tilde{x}}_{3}(t)$ is already very close to the exact solution $\bar{x}(t)=t$ of (49). In fact the plot of ${\tilde{x}}_{3}(t)$ in Figure 4 is visually indistinguishable from the plot of $\bar{x}(t)=t$. 

### 4.3. Fractional Variational Calculus of Variable Order

We now exemplify how the expansions obtained in Section 3 are useful to approximate solutions of fractional problems of the calculus of variations [21]. The fractional variational calculus of variable order is a recent subject under strong current development [15, 16, 22, 23]. So far, only analytical methods to solve fractional problems of the calculus of variations of variable order have been developed in the literature, which consist in the solution of fractional Euler-Lagrange differential equations of variable order [15, 16, 22, 23]. In most cases, however, to solve analytically such fractional differential equations is extremely hard or even impossible, so numerical/approximating methods are needed. Our results provide two approaches to this issue. The first was already illustrated in Section 4.2 and consists in approximating the necessary optimality conditions proved in [15, 16, 22, 23], which are nothing else than fractional differential equations of variable order. The second approach is now considered. Similar to Section 4.2, the main idea here is to replace the fractional operators of variable order that appear in the formulation of the variational problem by the corresponding expansion of Section 3, which involves only integer-order derivatives. By doing it, we reduce the original problem to a classical optimal control problem, whose extremals are found by applying the celebrated Pontryagin maximum principle [24]. We illustrate this method with a concrete example. Consider the functional

(54) $$\begin{array}{c} J\left(x\right)=\int_{0}^{1}{\left[{}_{0}{𝔻}_{t}^{\alpha (t)}x(t)-\frac{1}{\Gamma ((7-t)/4)}t^{(3-t)/4}\right]}^{2}dt, \end{array}$$

with fractional order *α*(*t*) = (*t* + 1)/4, subject to the boundary conditions

(55) $$\begin{array}{c} x\left(0\right)=0,\;\;x\left(1\right)=1. \end{array}$$

Since *J*(*x*) ≥ 0 for any admissible function *x* and taking $\bar{x}(t)=t$, which satisfies the given boundary conditions (55), gives $J(\bar{x})=0$, we conclude that $\bar{x}$ gives the global minimum to the fractional problem of the calculus of variations that consists in minimizing functional (54) subject to the boundary conditions (55). The numerical procedure is now explained. Since we have two boundary conditions, we replace _0_ *𝔻*_*t*_^*α*(*t*)^ *x*(*t*) by the expansion given in Theorem 7 with *n* = 1 and a variable size *N* ≥ 2. The approximation becomes

(56)   0𝔻tα(t)x(t)≈A(α(t),N)t−α(t)x(t) +B(α(t),N)t1−α(t)x(1)(t) +∑k=2NC(α(t),k)t1−k−α(t)Vk(t).

Using (56), we approximate the initial problem (54)-(55) by the following one: to minimize

(57) J~(x)=∫01[A(α(t),N)t−α(t)x(t)+B(α(t),N)t1−α(t)x(1)(t)+∑k=2NC(α(t),k)t1−k−α(t)Vk(t)−1Γ((7−t)/4)t(3−t)/4]2dt

subject to

(58) Vk(1)(t)=(k−1)tk−2x(t), Vk(0)=0,k=2,…,N,x(0)=0,  x(1)=1,

where *α*(*t*) = (*t* + 1)/4. This dynamic optimization problem has a system of ordinary differential equations as a constraint, so it is natural to solve it as an optimal control problem. For that, define the control *u* by

(59) u(t)=A(α(t),N)t−α(t)x(t)+B(α(t),N)t1−α(t)x(1)(t)+∑k=2NC(α(t),k)t1−k−α(t)Vk(t).

We then obtain the control system

(60) x(1)(t)=B−1tα(t)−1u(t)−AB−1t−1x(t)−∑k=2NB−1Ckt−kVk(t):=f(t,x(t),u(t),V(t)),

where, for simplification,

(61) $$\begin{array}{c} A=A\left(\alpha \left(t\right),N\right),\\ B=B\left(\alpha \left(t\right),N\right),\\ C_{k}=C\left(\alpha \left(t\right),k\right),\\ V\left(t\right)=\left(V_{2}\left(t\right),\ldots ,V_{N}\left(t\right)\right). \end{array}$$

In conclusion, we wish to minimize the functional

(62) $$\begin{array}{c} \tilde{J}\left(x,u,V\right)=\int_{0}^{1}{\left[u(t)-\frac{1}{\Gamma ((7-t)/4)}t^{(3-t)/4}\right]}^{2}dt \end{array}$$

subject to the first-order dynamic constraints

(63) x(1)(t)=f(t,x,u,V),Vk(1)(t)=(k−1)tk−2x(t), k=2,…,N,

and the boundary conditions

(64) x(0)=0,x(1)=1,Vk(0)=0, k=2,…,N.

In this case, the Hamiltonian is given by

(65) H(t,x,u,V,λ)=[u−1Γ((7−t)/4)t(3−t)/4]2+λ1f(t,x,u,V)+∑k=2Nλk(k−1)tk−2x

with the adjoint vector *λ* = (*λ*_1_, *λ*_2_,…, *λ*_*N*_) [24]. Following the classical optimal control approach of Pontryagin et al. [24], we have the following necessary optimality conditions:

(66) $$\begin{array}{c} \frac{\partial H}{\partial u}=0,\;\;x^{\left(1\right)}=\frac{\partial H}{\partial \lambda_{1}},\\ V_{p}^{\left(1\right)}=\frac{\partial H}{\partial \lambda_{p}},\;\;\lambda_{1}^{\left(1\right)}=-\frac{\partial H}{\partial x},\\ \lambda_{p}^{\left(1\right)}=-\frac{\partial H}{\partial V_{p}}. \end{array}$$

That is, we need to solve the system of differential equations

(67) x(1)(t)=B−1Γ((7−t)/4)−12B−2t2α(t)−2λ1(t)−AB−1t−1x(t)−∑k=2NB−1Ckt−kVk(t),Vk(1)(t)=(k−1)tk−2x(t), k=2,…,N,λ1(1)(t)=AB−1t−1λ1−∑k=2N(k−1)tk−2λk(t),λk(1)(t)=B−1Ckt−kλ1, k=2,…,N,

subject to the boundary conditions

(68) x(0)=0, Vk(0)=0, k=2,…,N,x(1)=1, λk(1)=0, k=2,…,N.

**Figure 5:**
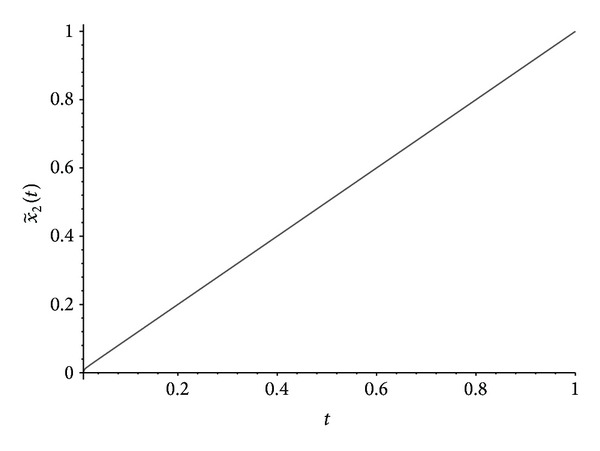
Approximation ${\tilde{x}}_{2}(t)$ to the exact solution $\bar{x}(t)=t$ of the fractional problem of the calculus of variations (54)-(55), obtained from the application of Theorem 7 and the classical Pontryagin maximum principle, that is, obtained by solving (67)-(68) with *N* = 2.

Figure 5 plots the numerical approximation ${\tilde{x}}_{2}(t)$ to the global minimizer $\bar{x}(t)=t$ of the variable order fractional problem of the calculus of variations (54)-(55), obtained by solving (67)-(68) with *N* = 2. The approximation ${\tilde{x}}_{2}(t)$ is already visually indistinguishable from the exact solution x¯(t)=t, and we do not increase the value of *N*. The effectiveness of our approach is also illustrated in Table 2, where some numerical values of the approximation ${\tilde{x}}_{2}(t)$ are given.

## Figures and Tables

**Table 1:** Some numerical values of the solution ${\tilde{x}}_{3}(t)$ of (53) with *N* = 3, very close to the values of the solution $\bar{x}(t)=t$ of the fractional differential equation of variable order (49).

*t*	0.2	0.4	0.6	0.8	1
${\tilde{x}}_{3}(t)$	0.20000002056	0.40000004031	0.60000009441	0.80000002622	1.0000001591

**Table 2:** Some numerical values of the solution ${\tilde{x}}_{2}(t)$ of (67)-(68) with *N* = 2, close to the values of the global minimizer $\bar{x}(t)=t$ of the fractional variational problem of variable order (54)-(55).

*t*	0.2	0.4	0.6	0.8	1
${\tilde{x}}_{2}(t)$	0.1998346692	0.3999020706	0.5999392936	0.7999708526	1.0000000000
